# Discussion on the Papers of Drs. Taylor and McLain

**Published:** 1910-10

**Authors:** B. L. Wyman

**Affiliations:** Birmingham, Ala.


### DISCUSSION ON THE PAPERS OF DRS. TAYLOR AND McLAIN.

DR. B. L. WYMAN, BIRMINGHAM, ALA.

I am very glad indeed of this opportunity to meet with this Association. It has been my desire to be with you before, but have never been able to attend a meeting.

Pellagra is a subject attracting our attention. Dr. Searcy first called attention to this disease, when he reported a case at Mt. Vernon Hospital, Mobile, Ala.; since that time many have followed his example, and at the present time, the disease is being studied generally. I have had six cases in the last six years, four of whom have died. One chronic case, apparently recovered. Last case treated by transfusion, recovered. In stating these six cases, wish to say that all were females except one. This man was a chronic alcohol victim, who had heard of pellagra, and himself suggested the disease, which I later diagnosed as such. One case a colored woman from Aberdeen, Miss., chronic case who improved under treatment, and is now apparently well. One case, a white female, I saw only twenty-four hours before death; this case had been under treatment for some time, being a chronic case, and I was told the nervous symptoms had manifested themselves. The next case was a lady with the acute form of Pellagra, who died in a few days after I saw her; she had a peculiar eruption on her hands, being on both dorsal and palma surfaces. She had severe proctitis and vaginitis, and was in a state of acute insanity when I saw her. Often the cutaneous symptoms appear late. I remember one patient who had all the characteristic symptoms except the insanity.

Dr. Davis transfused the one patient of whom I spoke, and she is apparently well, and I would prefer his telling about this case.

Dr. H. R. Slack, LaGrange, Ga.

It has been my misfortune to have treated eight patients with pellagra, four of whom have died; two are well; two still under treatment. Six were females, two males. One man alcoholic, and like Dr. Wyman's patient, he diagnosed his case first. The first paper read mentioned trinity of symptoms, but the order of sequence has been a little different with me, the mental and nervous symptoms preceding the gastric symptoms. During this period all lost greatly in weight, after that, the gastro-intestinal symptoms set up, the emaciation continuing, the cutaneous symptoms were last. It has been my observation that as the dysentery increases, the mouth gets better, and when the mouth is worse, the dysentery decreases. Two of these reported cases I believe are well; one a lady from Savannah, who belonged to a wealthy family, had never been known to eat corn bread, who had all the symptoms of pellagra, beginning with melancholia. I prescribed working in the flower garden, and when she did, she found that the sun burned her hands, causing an eruption, and the work caused dysentery, so she had to discontinue this. I then put her on treatment, and at last accounts she was up and well, but her hands are still red. One patient had lost a sister with tuberculosis of the bowels, and I am sure that this sister had died of pellagra. I do not try to check the bowels, I give no opiates, but give large doses of bismuth, with salol and creosote.

Dr. J. D. S. Davis, Birmingham, Ala.

I have seen only two cases of pellagra; first case was dying. The second case, the result was satisfactory in all respects, this was the case of which Dr. Wyman spoke, in which the transfusion method was used. The second day after transfusion, the change was so decided that the false membrane in the mouth began to peel off, the diarrhoea was checked, blood practically normal, and patient was able to be up and about the ward. The transfusion was done with a Crile Tube. The injection was made in the left fore-arm. The patient was bled twenty ounces before making the injection of blood, at which time the blood pressure had reached 90. I transfused her until her blood pressure was 140, and that of her brother, who was the donor, had reached about 130. This bleeding of the patient was done to prevent dilatation of the heart, etc. As a rule, I do not use local anesthetic, but when I do, use only a little salt solution under the skin, as I prefer it to cocaine. In bleeding a patient be careful that your * * * is such that you can get a transfusion, for if you have not a good blood supply, you may lose your patient.

DR. H. P. COLE, MOBILE, ALA.

One great mistake, I think we all make in considering pellagra, is that it is new. But it occurred in the 17th century, and has probably been with us ever since.

I have treated over one hundred cases, fifteen of these by transfusion, but in no case except where everything else had failed, and nothing else could be done. One patient died three hours after operation. One case was transfused at Houston, Texas, and one at Tifton, Ga. Two other cases operated on the blood was inadequate, one of the two cases was that of the blood taken from her husband who was 74 years old. Of the fifteen cases transfused, only six died, but cannot say that the transfusion was the direct cause of the recovery of the other nine. Of the six dead, two may properly be thrown out, as the operation was unsuccessful. Transfusion is not suitable to all cases of pellagra, ten cases were sent to us for operation, and all of these got well without it. I think it might be well just here to speak of the disease, pellagraphobia, a disease caused by fear of pellagra. Transfusion is suitable only in extreme cases, and otherwise hopeless, but if done then by men of experience, will often give good results, if done promiscuously will more often result in failure. As to the treatment; hygenic surroundings and forced food, with mental stimulant will do wonders. The prophylactic treatment of which Dr. Taylor speaks is good.

DR. PETTY OF MEMPHIS, TENN.

I want to ask Dr. Cole why he thinks transfusion is not good in the early stages of the disease?

Dr. Cole in reply. Necessarily, there is a certain element of danger in transfusion, there is danger of dilatation of the heart, etc.; Dr. Davis has given a remedy for dilatation, by bleeding before transfusion. The main reason why it is not necessary in the early stages, guess they will get well without these operations. By the early stages I mean the case who has had recurrent diarrhoea for three springs, melancholia, etc.

DR. TAYLOR IN CLOSING.

I want to thank the gentlemen for their discussion, and especially thank them for bringing out the transfusion method so forcibly. Do want to ask the doctors in their cases of transfusion, where they got good results, if the donor had ever had pellagra. (One replied, "Yes, and both donor and patient relapsed.")

One doctor brought out the question of mouth symptoms. This I consider is brought about by the poison changing places of escape, when the mouth is better the poison goes to the bowels, and vice versa. Transfusion should be done only in the last stages, and then by experienced men, as there is always an element of danger in it, and some of us had better not try it.

